# The chain mediation effects of general self-efficacy and psychological resilience between physical activity and academic stress among Chinese adolescents

**DOI:** 10.3389/fpsyg.2025.1626157

**Published:** 2025-08-01

**Authors:** Haokai Leng, Xianlin Xiang, Shihong Li

**Affiliations:** School of Physical Education, Shanghai University of Sport, Shanghai, China

**Keywords:** physical activity, academic stress, general self-efficacy, psychological resilience, chain mediation effect

## Abstract

**Introduction:**

Excessive academic stress is a significant factor influencing the mental health of Chinese adolescents. This study aimed to examine the relationship between physical activity and academic stress and explore the chain-mediated role of general self-efficacy and psychological resilience.

**Methods:**

A survey was administered to 1,230 adolescents using the Physical Activity Questionnaire (PAQ-A), the Academic Stress Scale, the General Self-Efficacy Scale (GSES), and the Psychological Resilience Scale.

**Results:**

(1) Adolescents' physical activity was negatively correlated with academic stress and positively correlated with general self-efficacy and psychological resilience; general self-efficacy was positively correlated with psychological resilience and negatively correlated with academic stress, while psychological resilience was negatively correlated with academic stress. (2) Physical activity directly affected adolescents' academic stress (β = 0.513, accounting for 64.52% of the total effect) and also indirectly influenced it through three pathways: Physical activity → general self-efficacy → academic stress (β = 0.058, 11.31% of total effect), Physical activity → psychological resilience → academic stress (β = 0.027, 5.26% of total effect), and Physical activity → general self-efficacy → psychological resilience → academic stress (β = 0.098, 19.10% of total effect).

**Discussion:**

Physical activity can directly influence adolescents' academic stress and indirectly affect it through the separate and chain mediation of general self-efficacy and psychological resilience.

## Introduction

Adolescence is a critical stage for the development of physical, cognitive, and emotional capabilities, and the accumulation of health during this period has a profound impact on an individual's life (Patton et al., 2016). Globally, approximately one in seven adolescents aged 10 to 19 experiences a mental disorder (World Health Organization, 2024a). Adolescence is characterized by significantly elevated stress levels (Gunnar et al., 2009). The “educational stress hypothesis” further suggests that societal emphasis on education inevitably generates various school-based stressors (Högberg et al., 2020; West and Sweeting, 2003). Among these, academic stress has emerged as one of the most significant stressors impacting adolescents' mental health. Academic stress refers to students' perception that their academic demands exceed their internal and external coping abilities (Walburg, 2014). In pursuit of excellent academic performance, adolescents are often under high academic stress (Yu et al., 2016). Chronic and excessive academic stress significantly harms adolescents' wellbeing. It impairs academic performance, leading to decreased achievement, reduced motivation, and academic burnout (Sasikumar and Balaraman, 2019; Putwain et al., 2023; Veyis and Simşek, 2019). Furthermore, it contributes to diverse mental health issues, including depression, insomnia, mood swings, irritability, test anxiety, and aggressive behaviors (Kristensen et al., 2023; Santiago et al., 2017; Sigfusdottir et al., 2016). Consequently, conducting comprehensive research on adolescent academic stress and developing effective interventions is critical.

It is widely accepted that physical activity promotes both physical and mental health. Physical activity has been shown to alleviate stress, improve symptoms of depression, reduce anxiety, and prevent cognitive decline (Stults-Kolehmainen and Sinha, 2014). Despite extensive evidence supporting the benefits of physical activity, three-quarters of adolescents fail to meet the World Health Organization's global physical activity guidelines (World Health Organization, 2024b). Within test-based education systems, adolescents commonly exhibit low physical activity levels combined with high sedentary time. This pattern of inactivity represents a significant public health concern. Research on 24-h activity patterns indicates that increased sedentary time is often coupled with reduced physical activity. This combination may be a key factor exacerbating the well-established challenge of academic stress in adolescents. However, research specifically examining the relationship between physical activity and academic stress within the adolescent population remains limited. Therefore, this study aims to investigate the relationship between physical activity, academic stress, general self-efficacy, and resilience in adolescents, and to identify the unique benefits of physical activity in alleviating academic stress, ultimately promoting adolescent mental health.

## Literature review and research hypotheses

### The influence of physical activity on academic stress

Physical activity is defined as any bodily movement produced by skeletal muscles that results in energy expenditure (Caspersen et al., 1985). The theory of the emotional effects of physical activity suggests that it ameliorates negative emotions such as stress, depression, and anxiety (Anderson and Brice, 2011; Ruby et al., 2011). Academic stress, a specific form of psychological stress, is a common stressor in school life and contributes significantly to negative emotions in adolescents. Physical activity is an effective means of reducing academic stress. A longitudinal study found that regular participation in physical activity during exam periods mitigated the negative effects of academic stress on students (Wunsch et al., 2017). Furthermore, a Spanish study found that adolescent boys were more physically active than girls and experienced lower academic stress (Galeano-Rojas et al., 2024). However, in China, adolescents face greater academic burdens and exhibit higher levels of sedentary behavior and lower physical activity. Greenberger et al. noted that the association between academic stress and poor mental health is stronger in Chinese adolescents than in students from other countries (Greenberger et al., 2000). This led to Hypothesis 1: Physical activity negatively predicts academic stress in adolescents.

### The mediating effect of general self-efficacy

General self-efficacy refers to an individual's belief in their ability to achieve goals or complete tasks (Bandura, 1978). Literature indicates that influences on general self-efficacy include prior experiences, social persuasion, and emotional and physiological states (Evans, 1989). Adolescents‘ sense of progress through personal effort, increased self-control, and peer encouragement during physical activity can enhance general self-efficacy. Studies in China (Zhang et al., 2024) and the United States (Joseph et al., 2014) show that physical activity promotes general self-efficacy. A review study found that general self-efficacy serves as both a determinant and an outcome of physical activity participation (McAuley and Blissmer, 2011). Critically, there is a strong association between general self-efficacy and stress. Social cognitive theory posits that an individual's judgment of the external environment influences emotional responses and cognitive processes (Bandura, 1997). Individuals with high general self-efficacy are resilient to external demands and confident in their development, making them more likely to view challenges as opportunities rather than obstacles (Li et al., 2018). Consistent with this, a study tracking adolescents' perceived stress, sleep quality, and general self-efficacy over two school weeks found that general self-efficacy had a positive ameliorative effect on stress (Ten Brink et al., 2021). This led to Hypothesis 2: General self-efficacy mediates the relationship between physical activity and academic stress.

### The mediating effect of psychological resilience

Psychological resilience, a central concept in positive psychology, refers to an individual's ability to adapt to adversity, trauma, tragedy, threats, or other major life stressors, reflecting the “ability to bounce back” from life's challenges and setbacks (Tsai and Freedland, 2022). Critically, resilience is not merely a genetic trait but rather a dynamic process and acquired characteristic that can be taught and developed (Joyce et al., 2018). Importantly, regular participation in physical activity has been shown to enhance perseverance and resilience in the face of adversity. For instance, Vella et al. conducted an intervention with 350 adolescents through organized physical activity and found that physical activity effectively enhanced psychological resilience in adolescents (Vella et al., 2021). Further supporting this link, existing literature suggests that adolescents enhance their resilience by meeting recommended physical activity levels (Gerber et al., 2012), maintaining the intensity at moderate-to-vigorous physical activity (Yu and Ye, 2023) and participating in team-based exercise (Hjemdal et al., 2006). Beyond its development through physical activity, psychological resilience serves a critical buffering role in reducing adverse emotions such as stress, anxiety, and depression. Liu et al. (2024) found that a supportive sports environment and interest in sports alleviated academic stress through psychological resilience in college students. Similarly, Resilience plays a crucial role in overcoming academic-related stress, with adolescents possessing high psychological resilience being less affected by academic stress than those with low resilience (Liu et al., 2022). Therefore, we propose Hypothesis 3: Psychological resilience mediates the relationship between physical activity and academic stress.

### The chain mediating effect of general self-efficacy and psychological resilience

General self-efficacy and psychological resilience are recognized as key psychological resources when individuals face stress, depression, and anxiety (Peñacoba et al., 2021). Crucially, these resources are interrelated. Specifically, general self-efficacy is considered an important internal protective factor within the cognitive dimension of the psychological resilience framework (Wang et al., 2018), it significantly contributes to the development and maintenance of psychological resilience. Individuals with high general self-efficacy tend to have higher confidence, better emotional regulation, and positive coping styles, making them more resilient to stress. In short, individuals with high general self-efficacy are likely to exhibit higher resilience. Therefore, based on the preceding evidence, Hypothesis 4 proposes that physical activity reduces adolescents' academic stress via enhanced general self-efficacy and psychological resilience.

**Figure 1:**
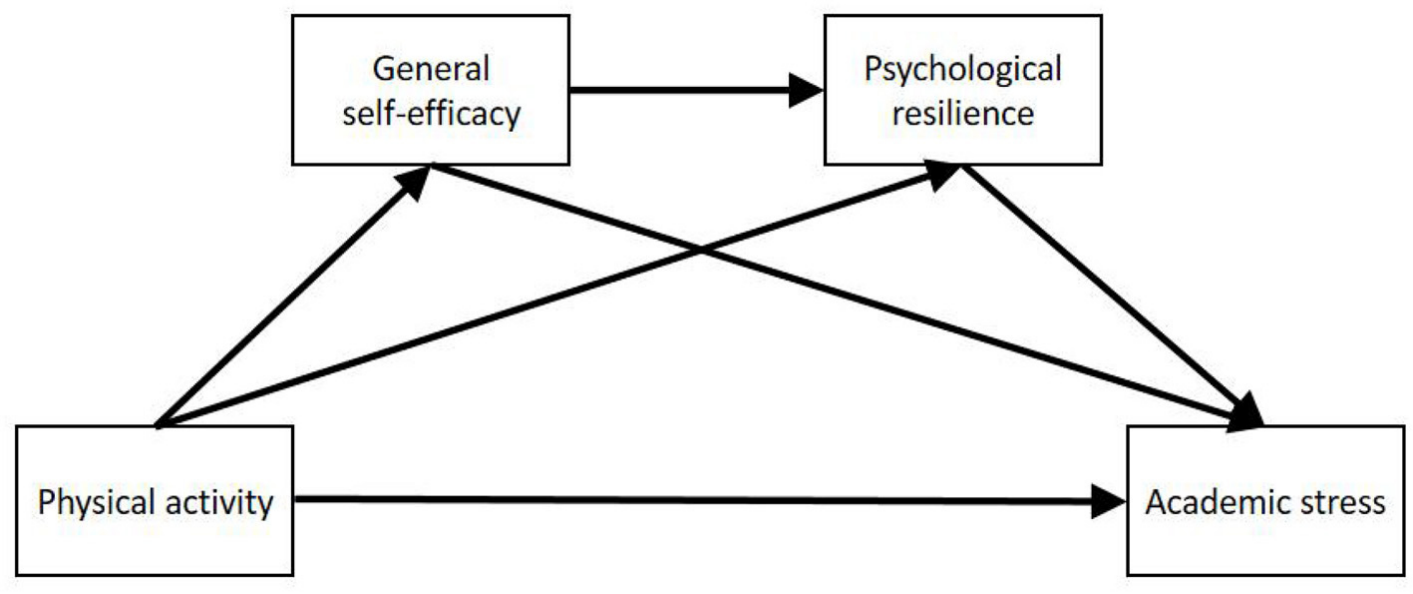
Chain mediation hypothesis model.

Collectively, this study proposes four research hypotheses and constructs a chain mediation model, as shown in Figure 1:

H1: Physical activity negatively predicts academic stress in adolescents.
H2: General self-efficacy mediates the relationship between physical activity and academic stress.
H3: Psychological resilience mediates the relationship between physical activity and academic stress.
H4: General self-efficacy and psychological resilience act as chain mediators between physical activity and academic stress.

## Methodology

### Data sources

This study was conducted between December 2024 and January 2025 in junior high schools in Shanghai, China, using class-based questionnaires. A convenience sampling method was employed to survey three districts in Shanghai (Yangpu, Xuhui, and Pudong). From each district, two schools were selected, and from each grade (first, second, and third) of these schools, two classes were chosen. Inclusion criteria: ① Junior high school students aged 12 to 15 years; ② No diagnosis of serious physical conditions (e.g., motor dysfunction, chronic diseases) or mental disorders (e.g., depression, anxiety); ③ Ability to independently read and comprehend the questionnaires in Chinese; ④ Students and their legal guardians must have signed a written informed consent form and voluntarily agreed to participate. Exclusion criteria: ① Inability to engage in regular physical activity during the previous week due to special circumstances or illness.; ② Absence from school due to special circumstances or illness; ③ Experiencing a major stressful event in the past 6 months (e.g., death of a family member, parental separation, serious accidents); ④ Incomplete questionnaire responses (e.g., more than 20% of questions unanswered) or clear logical inconsistencies (e.g., repeating the same question with identical extreme answers). Following the acquisition of informed consent from both students and their parents, 1,297 adolescents were recruited. The survey was administered using paper questionnaires, which were distributed and collected by investigators in collaboration with physical education teachers or classroom administrators. Of the 1,297 questionnaires distributed, 1,260 were returned (37 unaccounted for). After excluding 30 questionnaires with missing data, clear logical errors, or excessive answer repetition, 1,230 valid questionnaires were retained, resulting in a valid response rate of ~95%.

### Research tools

The overall questionnaire of this study consisted of basic information and four scales. The basic information consisted of gender (male, female), grade level (first year, second year, third year), academic performance (good, fair, poor) and their family's economic level (high-income family, middle-income family, low-income family).

#### Physical activity questionnaire for adolescents

Physical activity was assessed using the Physical Activity Questionnaire (PAQ-A), which asked adolescents about their participation in various types of physical activity during the previous week, including activity levels in physical education classes, lunch breaks, after school, and on weekends. Each question had five response options, ranging from “low” to “high” on a 1–5 scale. The total score was calculated by summing the scores of the nine questions and then averaging them, with higher scores indicating greater physical activity. Scores were categorized as follows: low physical activity level (score ≤ 2), medium physical activity level (2 < score < 3), and high physical activity level (score ≥ 3) (Qiang, 2016). After reliability testing, Cronbach's α for this scale in the current study was 0.829.

#### Academic stress scale for secondary school students

Academic stress in adolescents was measured using the “Academic Stress Scale for Secondary School Students,” developed by Jiajun et al. (2010). The questionnaire includes six items on parental stress, six on self-stress, five on teacher stress, and four on social stress. Each question offers five response options, ranging from “very inconsistent” to “completely consistent” on a 1–5 scale. The total score ranges up to 105, with higher scores indicating greater academic stress. After reliability testing, Cronbach's α for this scale in the current study was 0.891.

#### The general self-efficacy scale

The General Self-Efficacy Scale (GSES), the original version of which was developed by Schwarzer in 1981, was revised by Chinese scholar (Wang et al., 2001). The scale consists of 10 questions, each with four response options, ranging from “not at all correct” to “completely correct” on a 1–4 scale, with a maximum possible score of 40. Higher scores indicate better general self-efficacy. After reliability testing, Cronbach's α for this scale in the current study was 0.803.

#### The adolescent resilience scale

The Adolescent Resilience Scale, developed by Hu Yueqin et al., has been widely used in recent studies. The scale consists of 27 items, divided into two dimensions: personal strength and support (Yueqin and Yiqun, 2008). Each question offers five response options, ranging from “very compliant” to “very non-compliant,” corresponding to a score of 1–5. The total score is calculated by summing the individual scores, with higher scores indicating a higher level of resilience. After reliability testing, Cronbach's α for this scale in the current study was 0.857.

### Data analysis

Data analysis was conducted using SPSS 26.0 statistical software. First, Harman's one-factor test was applied to examine common method bias. Descriptive statistics were used for demographic analysis, followed by a one-way ANOVA to examine differences in general self-efficacy, psychological resilience, and academic stress across physical activity levels. Pearson's correlation was performed for correlation analysis, with gender, grade level, family economic status, and academic performance as control variables. Logistic regression was then used to analyze the relationships between physical activity, academic stress, general self-efficacy, and psychological resilience. Finally, the data were standardized, and Process 3.5 plugin, model 6, was used to test the chain mediation effect of physical activity, academic stress, general self-efficacy, and psychological resilience. Statistical significance was set at *p* < 0.05.

## Research results

### Common method bias

Non-rotated exploratory factor analysis was performed on all indicators using the Harman one-way test. The results showed that a total of 20 factors with eigenvalues >1 were obtained. Among them, the explained variance of the first factor was 15.38%, which was much lower than the critical value of 40%. It proves that there is no serious common method bias in this study.

### Sample characteristics

Table 1 presents the demographic characteristics of the sample. The sample consisted of 623 boys (50.70%) and 607 girls (49.30%). In terms of grade distribution, 470 adolescents (38.2%) were in the first year, 338 (27.5%) in the second year, and 422 (34.3%) in the third year. Regarding academic performance, the largest group was 483 adolescents (39.3%) with “average” performance, followed by 433 (35.2%) with “good” performance, and 314 (25.5%) with “poor” performance. In terms of family economic level, 437 adolescents (57.3%) came from “middle-income families,” followed by 337 (35.5%) from “high-income families,” and 88 (7.2%) from “low-income families.”

**Table 1:** Statistics on basic information of survey respondents (*n* = 1,230).

**Variable name**	**Categories**	**Frequency**	**Percentage**
Gender	Male	623	50.70%
	Female	607	49.30%
Grade	First year	404	32.80%
	Second year	404	32.80%
	Third year	422	34.30%
Academic performance	Good	433	35.20%
	Average	483	39.30%
	Poor	314	25.50%
Family economic level	High-income families	437	35.50%
	Middle-income families	705	57.30%
	Low-income families	88	7.20%

### Correlation analysis of the key variables

Pearson correlation analysis was conducted to examine the relationships among physical activity, academic stress, general self-efficacy, and resilience. The results, shown in Table 2, indicate the following: Physical activity was negatively correlated with academic stress (r = −0.541, *p* < 0.01), positively correlated with general self-efficacy (r = 0.487, *p* < 0.01), and positively correlated with psychological resilience (r = 0.297, *p* < 0.01). Academic stress was negatively correlated with general self-efficacy (r = −0.484, *p* < 0.01) and psychological resilience (r = −0.561, *p* < 0.01). General self-efficacy was positively correlated with psychological resilience (r = 0.545, *p* < 0.01).

**Table 2:** Descriptive statistics and correlation analysis of variables (*n* = 1,230).

**Variable name**	***M ±SD***	**PA**	**AS**	**GSE**	**PR**
PA	2.602 ± 0.735	1			
AS	52.810 ± 12.525	−0.541^***^	1		
GSE	25.470 ± 5.282	0.487^***^	−0.484^***^	1	
PR	94.240 ± 16.487	0.297^***^	−0.561^***^	0.545^***^	1

^*^p < 0.05.

^**^p < 0.01.

^***^p < 0.001.

PA, Physical activity; AS, Academic stress; GSE, General self-efficacy; PR, Psychological resilience.

To more clearly and accurately observe the effect of physical activity level on adolescents' academic stress, self-efficacy, and psychological resilience, a one-way ANOVA was conducted. The results in Table 3 indicate that academic stress decreased, while general self-efficacy and psychological resilience increased as physical activity level increased.

**Table 3:** ANOVA results of the effects of different levels of physical activity on academic stress, general self-efficacy and their psychological resilience in adolescents.

**Variable name**	**Low physical activity level**	**Moderate physical activity level**	**Higher physical activity levels**	**F**	**LSD**
AS	64.10 ± 9.44	51.63 ± 11.33	45.11 ± 10.49	226.27^***^	>>
GSE	21.32 ± 5.47	24.61 ± 5.50	28.43 ± 5.76	119.53^***^	>>
PR	86.64 ± 14.50	96.63 ± 16.23	101.57 ± 16.48	63.68^***^	>>

^*^p < 0.05.

^**^p < 0.01.

^***^p < 0.001.

, Low physical activity level; , Moderate physical activity level; , Higher physical activity levels.

PA, Physical activity; AS, Academic stress; GSE, General self-efficacy; PR, Psychological resilience.

### Hypothetical model testing

Regression analyses were conducted with psychological resilience as the dependent variable and grade level, gender, academic performance, and family economic level as control variables. Academic stress, general self-efficacy, and psychological resilience were used as dependent variables in the respective analyses. As shown in Table 4, physical activity negatively predicted academic stress in adolescents (β = −0.331, *p* < 0.001), while positively predicting general self-efficacy (β = 0.473, *p* < 0.001) and psychological resilience (β = 0.064, *p* < 0.05). General self-efficacy positively predicted psychological resilience (β = 0.490, *p* < 0.05) and negatively predicted academic stress (β = −0.123, *p* < 0.001). Psychological resilience negatively predicted academic stress (β = −0.420, *p* < 0.001).

**Table 4:** Regression analysis of the relationship between variables in the adolescent population.

**Regression equation**		**Overall fit index**			**Regression coefficient**	**Significance**
**Dependent variable**	**Independent variable**	**R2**	*Δ**R***	**F**	β	**t**
AS	Gender	0.313	0.240	111.436^***^	0.088	3.618^***^
	Grade				−0.021	−0.865
	Academic performance				0.088	3.563^***^
	Family economic level				0.037	1.481
	PA				−0.513	−20.675^***^
GSE	Gender	0.361	0.204	138.501^***^	0.209	8.876^***^
	Grade				−0.039	−1.669
	Academic performance				−0.266	−11.206^***^
	Family economic level				−0.077	−3.166^**^
	PA				0.473	19.78^***^
	Gender	0.304	0.154	88.821^***^	0.066	2.603^**^
	Grade				0.013	0.538
PR	Academic performance				−0.039	−1.477
	Family economic level				−0.027	−1.048
	PA				0.064	2.235^*^
	GSE				0.490	16.419^***^
AS	Gender	0.505	0.123	177.811^***^	0.185	8.605^***^
	Grade				−0.028	−1.37
	Academic performance				−0.016	−0.718
	Family economic level				0.001	0.040
	PA				−0.331	−13.623^***^
	GSE				−0.123	−4.402^***^
	PR				−0.420	−17.41^***^

^*^p < 0.05.

^**^p < 0.01.

^***^p < 0.001.

PA, Physical activity; AS, Academic stress; GSE, General self-efficacy; PR, Psychological resilience.

First, gender, grade level, academic performance, and family economic status were controlled. Second, the four variables of physical activity, general self-efficacy, psychological resilience and academic stress were standardized. Finally, the chain- mediation model was validated using the Process 3.5 plugin, employing the Bootstrap method with 5,000 samples and a 95% confidence interval. It is generally accepted that if the BootLLCI and BootULCI do not contain 0, the mediation effect is significant. Table 5 shows that physical activity has a total effect of −0.513 on academic stress. The direct effect of physical activity on academic stress is −0.331 (95% CI = −0.379 to −0.283), indicating a significant direct effect of physical activity on academic stress, accounting for 64.33% of the total effect. Thus, hypothesis H1 is supported.

**Table 5:** Bootstrap test for mediating effect.

**Total effect**	**β**	**BootSE**	**BootLLCI**	**BootULCI**	**%**
PA → AS	−0.513	0.025	−0.562	−0.465	100%
**Direct relationship**					
PA → AS	−0.330	0.024	−0.379	−0.283	64.33%
PA → GSE	0.473	0.024	0.043	0.520	-
PA → PR	0.064	0.029	0.008	0.121	-
GSE → PR	0.490	0.030	0.432	0.549	-
GSE → AS	−0.123	0.028	−0.177	−0.068	-
PR → AS	−0.420	0.024	−0.467	−0.373	-
**Indirect relationship**					
PA → GSE → AS	−0.058	0.016	−0.091	−0.026	11.31%
PA → PR → AS	−0.027	0.013	−0.051	−0.003	5.26%
PA → GSE → PR → AS	−0.098	0.010	−0.119	−0.078	19.10%

Each continuous variable in the model is standardized.

PA, physical activity; AS, Academic stress; GSE, General self-efficacy; PR, Psychological resilience.

**Figure 2:**
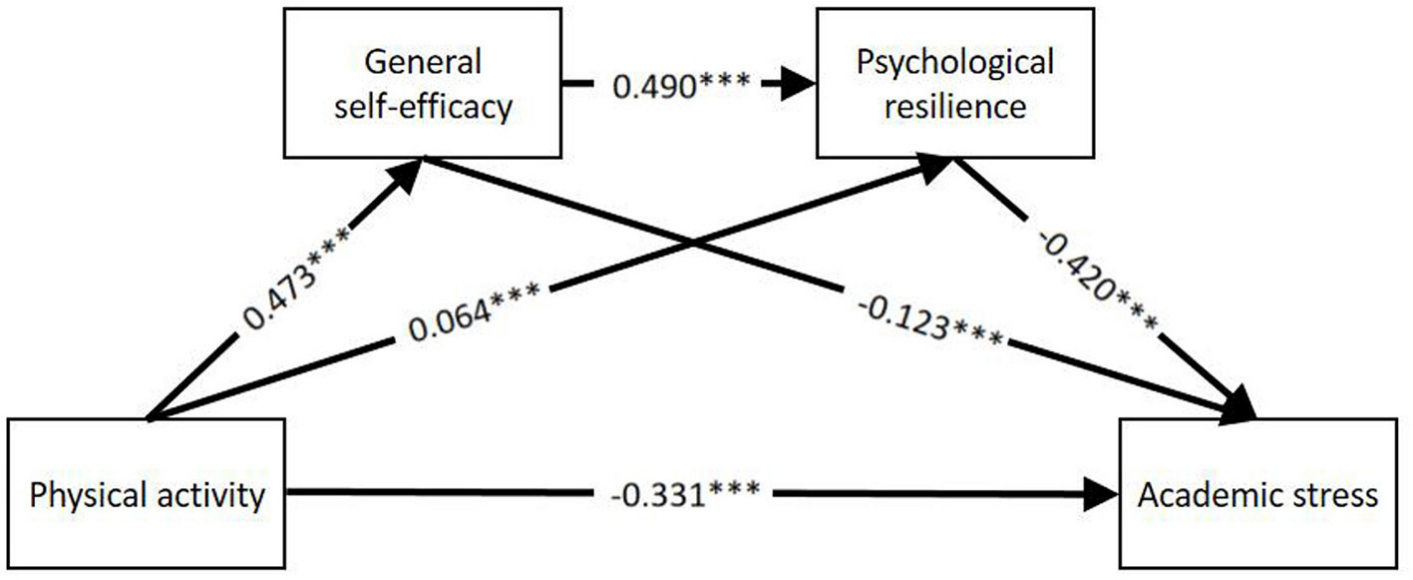
Chain mediation model of physical activity, general self-efficacy, psychological resilience and academic stress. ^*^p < 0.05, ^**^p < 0.01, ^***^p < 0.001.

From the model, it can be seen that physical activity predicts academic stress and that general self-efficacy and resilience play an indirect mediating utility in which there are three paths. Among them, the first mediating effect path: PA → GSE → AS, with an indirect effect value of −0.058, 95% CI = (−1.5423; −0.4464), which accounted for 11.31% of the total effect, and the hypothesis of H2 was valid. The second mediated effect path: PA → PR → AS, with an indirect effect value of −0.027, 95% CI = (−2.0273; −1.3291), accounting for 5.26% of the total effect, assuming that H3 holds. The third chain mediated effect pathway: PA → GSE → PR → AS, with an indirect effect value of −0.098, 95% CI = (−0.8706; −0.0536) accounted for 19.10% of the total effect, and hypothesis H4 was established. In summary it can be concluded that physical activity directly predicts academic stress, physical activity indirectly predicts academic stress through general self-efficacy, and physical activity also indirectly predicts academic stress through resilience. With the data from each pathway, Table 5 was converted into a chain-mediated model diagram, see Figure 2.

## Discussion

Excessive academic stress among adolescents poses a major challenge to their psychological wellbeing, making the identification of effective mitigation strategies a key research priority. This study demonstrates that physical activity exerts a significant influence on adolescents' academic stress through the chain mediation effect of general self-efficacy and psychological resilience. This finding provides a novel perspective on understanding how physical activity alleviates academic stress. Nevertheless, the questionnaire measures employed in this study may be subject to subjective bias, particularly recall bias in adolescents' self-reported physical activity levels over the preceding week. Future research could utilize accelerometers (e.g., wearable devices) to address this limitation. Additionally, longitudinal designs tracking adolescents' development over time are recommended to more accurately establish the causal relationships and temporal sequence within the identified chain mediation pathway.

### The influence of physical activity on academic stress

The results of this study indicate a significant negative correlation between physical activity and academic stress in adolescents. Physical activity negatively predicts academic stress, confirming hypothesis H1, which aligns with previous studies (Wunsch et al., 2017). The mechanism of action may be twofold, based on existing research findings. First, physical activity promotes the secretion of hormones such as endorphins and dopamine, which induce a sense of pleasure (Fiore et al., 2016; Harber and Sutton, 1984). Second, physical activity helps distract from and release negative emotions such as tension, worry, dissatisfaction, and anger, reducing adolescents' perception of academic stress. The physical activity guidelines for children and adolescents in Canada (Tremblay et al., 2016) and Australia (Anthony Okely et al., 2021) recommend at least 60 min of MVPA daily, with adherence to these guidelines promoting better mental health in adolescents (Carson, 2018). This study examined the relationship between physical activity levels and academic stress, finding that adolescents with low physical activity levels experienced higher academic stress, while those with moderate to high levels reported lower academic stress. This further supports the notion that higher levels of physical activity reduce academic stress. These results suggest that maintaining a high level of physical activity has a more significant impact on reducing academic stress in adolescents.

### The mediating effect of general self-efficacy

This study further confirmed the mediating role of general self-efficacy between physical activity and academic stress in adolescents. Specifically, physical activity not only directly alleviates academic stress but also indirectly reduces it by enhancing general self-efficacy, thus supporting hypothesis H2. This suggests that enhancing general self-efficacy is a key mechanism through which physical activity influences academic stress in adolescents. General self-efficacy is closely related to physical activity (Warner et al., 2014). When individuals engage in physical activity, mastering motor skills and achieving set goals through sustained practice boosts their confidence, thereby enhancing general self-efficacy. This aligns with Bandura's social learning theory, which posits that personal success experiences boost general self-efficacy. Exploring the relationship between physical activity levels and general self-efficacy reveals that adolescents with higher physical activity levels exhibit better general self-efficacy than those with lower levels. It is hypothesized that physical activity creates a positive feedback loop, enhancing adolescents' sense of self-control and fostering an internalized belief in their ability to overcome various challenges, thereby boosting general self-efficacy. Additionally, the appraisal theory of stress suggests that individuals respond to stress in two ways: challenging appraisals encourage constructive strategies and positive outcomes, while hindering appraisals provoke defensive strategies and negative outcomes (Folkman, 2013; Zhang et al., 2019). General self-efficacy does not diminish stressors themselves but influences an individual's perception of stress. A key source of stress arises when individuals feel unable to cope with challenges or difficulties. Previous research has shown that individuals with high general self-efficacy are more likely to use constructive coping strategies for stress (Hampel, 2007). Thus, adolescents with high general self-efficacy tend to perceive academic stress more accurately and have greater confidence in overcoming academic challenges. Based on the findings of this study, we hypothesize that when students engage in physical activity and build general self-efficacy, they may transfer this confidence to their academic pursuits, believing they can master the material and achieve their academic goals, thereby reducing academic stress.

### The mediating effect of psychological resilience

This study confirmed Hypothesis H3, demonstrating that psychological resilience serves as a mediator in the relationship between physical activity and academic stress in adolescents. Physical activity was found to positively predict adolescents‘ psychological resilience, which in turn mediates the relationship between physical activity and academic stress. This study found a positive correlation between physical activity levels and psychological resilience, consistent with prior research (Gerber et al., 2012). Physical activities like weight loss, muscle building, and improving athletic performance help regulate attention, enhance cognitive abilities, and improve emotional regulation—all factors that contribute to psychological resilience in adolescents. Adolescents experience academic stress from factors like parental expectations, teachers' discipline, and peer competition, all of which require strong pressure resilience. Psychological resilience reflects positive personality traits and psychological abilities, enabling individuals to maintain emotional stability under stress and effectively cope with life's challenges, making it a crucial psychological resource for personal growth (Cazan and Truta, 2015). Research has shown that psychological resilience not only helps secondary school students manage academic stress but also enables them to mobilize resources flexibly and adopt constructive coping strategies for academic challenges (Friedberg and Malefakis, 2018). Resilient adolescents tend to have stronger willpower and are less affected by negative events, leading to lower academic stress. This suggests that physical activity is a key pathway for enhancing psychological resilience, which in turn buffers the negative effects of academic stress.

### The chain mediating effect of general self-efficacy and psychological resilience

The study confirmed Hypothesis H4, establishing that general self-efficacy and psychological resilience exhibit a chain-mediating effect between physical activity and academic stress. These constructs represent key psychological resources for fostering positive psychological outcomes. To more clearly and accurately observe the effect of physical activity level on adolescents' academic stress, self-efficacy, and psychological resilience, a one-way ANOVA was conducted. The results (Table 3) indicate that academic stress decreased, while general self-efficacy and psychological resilience increased as physical activity level increased. The analysis of the chain mediation effect revealed that the effect size of the pathway “PA → GSE → PR → AS” was 0.098, accounting for 19% of the total effect. This suggests that physical activity can indirectly influence adolescents' academic stress through the chain mediation of general self-efficacy and psychological resilience. Previous research has shown that general self-efficacy and psychological resilience mediate the relationship between physical activity and life satisfaction (Deng et al., 2023). First, physical activity helps adolescents enhance their fitness and regulate emotions. It fosters a sense of accomplishment in overcoming challenges and achieving goals, which in turn enhances general self-efficacy and transfers to other areas of life and learning. Second, general self-efficacy is a recognized factor influencing psychological resilience (Wang et al., 2018). Individuals with high general self-efficacy are better equipped to cope with adversity and challenges, as they believe in their ability to overcome difficulties. Strong self-control beliefs enable individuals to persist through stressful and unfavorable situations, thereby enhancing resilience. This suggests that general self-efficacy is a crucial psychological resource that positively influences the formation and development of resilience. Finally, psychological resilience helps individuals cope effectively with stress and challenges, facilitates better adaptation to the learning environment, promotes a positive attitude toward academic challenges, protects students' interest and motivation in learning, and mitigates the negative effects of academic stress. The chain mediation model proposed in this study integrates research on the relationships between general self-efficacy, psychological resilience, and academic stress. It provides a more comprehensive understanding of how physical activity affects adolescents' academic stress and offers guidance for promoting reforms in school physical education and fostering adolescents' positive psychology.

## Limitations

This study has several limitations. First, the cross-sectional design of the study prevented the establishment of causal relationships between the variables. Second, the study relied on self-report measures, whereas the use of accelerometers to measure physical activity might have provided more accurate data. Finally, the study focused solely on adolescents in Shanghai, China, without considering other regions. Therefore, caution is necessary when generalizing these findings to other populations, and further studies with larger, more controlled samples are needed. Despite these limitations, the study offers valuable theoretical and practical insights.

## Conclusion

Adolescents' general self-efficacy and psychological resilience partially mediated the relationship between physical activity and academic stress. Additionally, general self-efficacy and resilience played a chain-mediation role between physical activity and academic stress. Specifically, physical activity directly influences adolescents' academic stress and also indirectly affects it through general self-efficacy and resilience.

## Data Availability

The original contributions presented in the study are included in the article/supplementary material, further inquiries can be directed to the corresponding author.
